# *Caenorhabditis elegans* Maintains Highly Compartmentalized Cellular Distribution of Metals and Steep Concentration Gradients of Manganese

**DOI:** 10.1371/journal.pone.0032685

**Published:** 2012-02-29

**Authors:** Gawain McColl, Simon A. James, Sheridan Mayo, Daryl L. Howard, Christopher G. Ryan, Robin Kirkham, Gareth F. Moorhead, David Paterson, Martin D. de Jonge, Ashley I. Bush

**Affiliations:** 1 Mental Health Research Institute, University of Melbourne, Parkville, Australia; 2 Centre for Neuroscience, University of Melbourne, Parkville, Australia; 3 Commonwealth Scientific and Industrial Research Organisation, Clayton, Australia; 4 Australian Synchrotron, Clayton, Australia; 5 Department of Pathology, University of Melbourne, Parkville, Australia; Rosalind Franklin University, United States of America

## Abstract

Bioinorganic chemistry is critical to cellular function. Homeostasis of manganese (Mn), for example, is essential for life. A lack of methods for direct *in situ* visualization of Mn and other biological metals within intact multicellular eukaryotes limits our understanding of management of these metals. We provide the first quantitative subcellular visualization of endogenous Mn concentrations (spanning two orders of magnitude) associated with individual cells of the nematode, *Caenorhabditis elegans*.

## Introduction

Inorganic co-factors are fundamental to biological systems. Despite this vital role of inorganic chemistry in biology, and recent advances in the tools available for metalloprotein bioinformatics [1], [2], it remains difficult to understand the interactions between metals and cellular macromolecules that define an organism's biochemistry. Approximately one half of known protein structures contain a metal cofactor [1], [3]. Metal dependent metabolism is involved in a myriad of chemical processes including efficient energy maintenance, catalysis under physiological conditions, and highly selective stereo-synthesis. An understanding of protein structure and function requires knowledge of metal-macromolecular interactions.

Manganese (Mn) is an essential cofactor for multiple classes of enzymes, e.g. transferases, hydrolases, lyases, and superoxide dismutase (SOD). The reduction of molecular oxygen in all aerobic cells results in intermediates such as superoxide radicals, hydrogen peroxide and hydroxyl ions, which are highly toxic and may contribute to biological senescence [4]. Typically, these species are decomposed by a cells complement of antioxidants, of which Mn-SOD plays an important role in reducing the oxidative burden.

Mn uptake requires orthologs of mammalian divalent metal transporters (DMT1) and yeast Smf proteins, which belong to the wider family of natural resistance-associated macrophage protein (NRAMP) [5]. In *C. elegans*, loss-of-function alleles of the three *smf* genes alter toxicity to excess Mn [5], [6]. Interestingly, these mutants also show decreased innate immunity suggesting transport of Mn from the intestinal lumen limits colonization by potential microbial pathogens. However, how and where intracellular Mn is stored within a multicellular animal remains unknown.

*C. elegans* is a widely adopted model system in biological studies, making this multicellular organism attractive for the study of inorganic elements and biological-metal distribution, including homeostatic maintenance. The optical transparency, relatively simple anatomy, defined cell lineage, and well-characterized genetics have made *C. elegans* a proven tool for developmental studies of protein localization and function. Until now, studies of the inorganic elements intrinsic to protein structure and function have lacked methods for direct *in situ* quantitation and visualization. Whilst traditional bulk analysis techniques (e.g. inductively coupled plasma spectroscopy) possess formidable sensitivity, they are destructive and provide information on total elemental abundances only. Histochemical staining has been utilized for localizing metals in cellular structures but such methods have significant limitations including issues with specificity (for example, no stains are specific for Mn), difficulty with simultaneous analysis of multiple elements, and the lack of quantitative data.

The establishment of detailed anatomical maps that define Mn and other bioinorganic cellular and subcellular distribution is essential to build our biological understanding. Furthermore, such data would provide reference points to protein-based studies of metal homeostasis. Scanning x-ray fluorescence microscopy is ideally suited for investigating elemental distributions within biological specimens [7]. However, the typically slow acquisition rates and poor detector efficiency of these instruments has limited the application of this technique to complex biological systems [8]. High-resolution studies of larger, intact and complex biological specimens, such as *C. elegans*, have been impossible due to sample aging and radiation damage.

Furthermore, as the incident beam excites elemental fluorescence from the full thickness of the specimen, the resulting 2D images display projected elemental content, and unambiguous interpretation of elemental localization therefore remains difficult.

## Results and Discussion

We therefore took advantage of developments in tomographic analysis to further identify features not readily discernible in 2-D [9]. In this study we use a 96-channel silicon detector system (Maia) capable of event-mode x-ray fluorescence detection [10]. This detector accelerated measurements by over two orders of magnitude to not only quantitate elemental composition but also interrogate the detailed subcellular spatial arrangement of elements *in situ* in whole, unsectioned adult *C. elegans*. A 100-projection rotation series was obtained over 360 degrees in 5 hours with a dose of ∼10 MGy. Each projection was generated with an effective dwell time of 3 ms per 1.6 µm^2^ pixel. The x-ray fluorescence data, collected over 8 megapixels of exposure, represent an order of magnitude more information than any previous x-ray fluorescence tomography measurements [11], [12]. Individual elemental maps were combined to generate a tomographic visualization of the anterior two-thirds (650 µm) portion of the specimen with an estimated resolution of ∼7 µm^3^.

**Figure 1:**
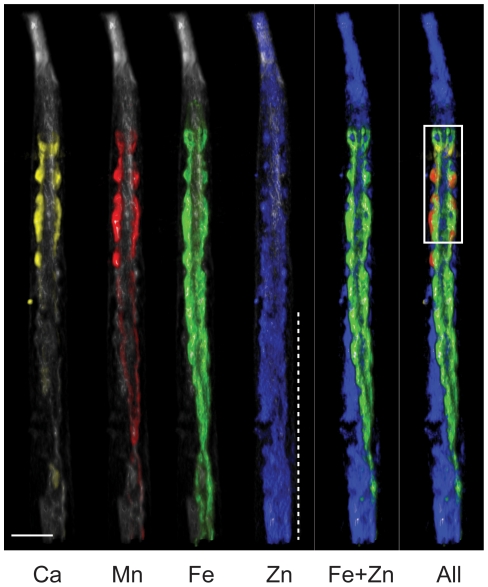
2D projection of elemental tomography in a lyophilized-wild type adult *C. elegans*. Shown are K (white), Ca (yellow), Mn (red), Fe (green), Zn (blue) and elements in combination. White box defines region examined in close-up (Fig. 2). Dashed line indicates the gonad. Bar = 50 µm. A movie of the reported elements is shown in Movie S1.

**Figure 2:**
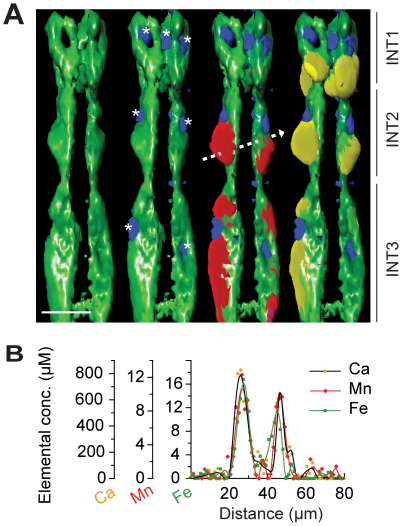
Surface rendered close-up and concentration plot of elements in the anterior intestinal (INT) cells. (A) Surface rendering shows Fe (green) is restricted to the cytoplasm of the INT cells, and excluded from nuclei. Zn (blue) is concentrated within nuclei (* = nuclei). Mn (red) and Calcium (yellow) show subcellular enrichment. Dashed line represents transect examined in panel B. INT cells marked as labeled. Bar = 25 µm. (B) Plot of elemental (Ca, Mn and Fe) concentration extracted from transect through the intestinal region.

The distributions of inorganic elements (K to Zn) are shown in Figure 1 (for stereo image see Figure S1 and Movie S1). The nematode intestine is comprised of a ring of four epithelial cells (INT1) immediately posterior to the pharynx, and subsequent pairs of cells moving to the posterior (INT2 through INT9). The INT cells show distinct Ca and Mn localization. To more closely examine the anterior intestine we used surface rendering to highlight cellular structures (Figure 2). A single 2-D projection was chosen and the elemental concentrations determined along a transect line through the INT2 cell pair and the intestinal lumen (dashed line Figure 2a). The concentrations of Ca, Mn and Fe along this line were plotted (Figure 2b). Mn present in the INT2 cell pair is enriched ∼450-fold compared to the intestinal lumen. The peak Mn concentration within the entire sample was mapped to the INT2 cell pair at 38 µM. How the intestinal cells safely maintain such high Mn concentration under normal physiological conditions remains unknown. Exogenous Mn above 75 µM reduces body and brood size, and median life span in the nematode [13], while as little as 5 µM is toxic to primary cells in culture [14]. The maintenance of this steep Mn concentration gradient implies a critical role for this metal in the intestine. Uptake and sequestration of Mn from the intestinal lumen may be important for innate immunity, as Mn is essential for bacterial virulence [15].

In the *C. elegans*, intestine intracellular Ca concentration is dynamic and oscillates to regulate peristalsis [16], [17]. In our sample this flux in Ca has been captured, such that the anterior intestinal cells contained the peak Ca concentration of ∼3.2 mM (INT1 cell pair). The distribution of both Ca and Mn in the intestinal cells is not homogeneous, and appears to overlap (particularly in INT2 and INT3, Figure 2). This region may include endoplasmic reticulum, which is thought to be involved in Ca storage and is abundant in intestinal cells [18].

The intestine is also rich in Fe and shows a characteristic left-handed twist along the longitudinal axis as it negotiates around the nematode gonad (Figure 1). The peak Fe concentration of 26 µM was found in the INT3 cells. We observed that, although the intestinal cell cytoplasm is rich with Fe, this element is excluded from intestinal nuclei (Figure 1 and 2). In contrast, Zn is concentrated within these large nuclei, conceivably as a cofactor for nuclear transcription factors. Zn also has a wider distribution, consistent with other nuclei, including those of the nerve ring, gonad and developing embryos (see movie S1).

Studies of individual purified proteins, recombinant proteins or isolated biological fluids [1], [19] can be confounded by the incorrect metallation or even inadvertent exclusion of the relevant metal species altogether [20], [21]. Furthermore, sample preparation, in particular the use of chemical crosslinking prior to analysis, can adversely affect metal measures [22]. These considerations emphasize the importance of minimally invasive sample treatment to maintain the *in vivo* status of metals. Recent work has highlighted the diversity and complexity of the prokaryotic metallome and exposed our limited understanding of how metalloprotein interactions influence microbial physiology [23]. A deeper understanding of inorganic physiology in more complex eukaryotic systems is increasingly necessary to tease apart the critical roles metals play in development, health and disease.

Our investigations indicate that Mn and other elements are strictly maintained and enriched within specific cell types of *C. elegans*. Direct *in situ* mapping of these elements at the sub cellular level in this multicellular system has not previously been achieved. Having determined the wild type elemental distribution in young adult *C. elegans*, the wealth of mutants and disease models for this organism can now be exploited to explore the role of biological metal homeostasis in development and disease.

## Materials and Methods

### Strains

Wild-type Bristol strain (N2) was provided by the *Caenorhabditis* Genetics Center and cultured at 20°C under standard conditions [24].

### Scanning x-ray Fluorescence Tomography

A cohort of developmentally synchronous 4-day old adults was then washed four times in excess of S-basal [24] to remove excess bacteria, anesthetized in ice-cold 0.2% (w/v) NaN_3_, then washed again in ice-cold 1.5% (w/v) CH_3_COONH_4_. Individuals were then deposited onto a silicon nitride window (Silson). Excess buffer was removed via fine tapered paper wicks (MiTeGen) and the samples straightened using an eyelash. The window was then frozen in liquid N_2_-chilled liquid propane using a KF-80 plunge freezer (Leica) and lyophilized overnight at −40°C. Immediately following drying the straightest sample was then removed (via an eyelash) and sandwiched, by its tail, between two pieces of adhesive tape backed onto a small tab of developed autoradiography film and mounted for tomographic imaging.

A beam of 10-keV x-rays was focused to a spot of ∼1.5-µm diameter using a Kirkpatrick-Baez mirror pair at the X-ray Fluorescence Microscopy beamline of the Australian Synchrotron [25]. The sample was scanned through the focus at constant velocity (0.6 mm/s) using a 96-channel Maia detector system [10], which recorded the x-rays emitted into a 0.2-steradian solid-angle cone orientated at 90° to the incident beam. Real-time processing of the recorded x-ray events was processed using the Maia field programmable gate array (FPGA), and these x-ray events (characterized by energy, time-over-threshold, and detector identity) were streamed to disk. Pixel boundary transitions were defined by interleaving the scan stage positions (horizontal and vertical) at 1.25 µm intervals with the x-ray events. Spectral deconvolution and imaging were performed using the Dynamic Analysis method [26] and GeoPIXE software (http://nmp.csiro.au/GeoPIXE.html). This analysis reduced the data to 100, 2-D projected images of the distributions of 12 elements (K - Zn).

### Tomographic analysis

The projected images were aligned 1) vertically, using cross-correlation technique and 2) horizontally, using the ‘centre-of-mass’ of the fluorescence signal. The Fe image sequence had the most well defined features, and so was used to determine the sequence of image shifts that were required to align the dataset. These shifts were then applied to all projections so as to align the images for all elements.

X-TRACT (http://xrsi.cmit.csiro.au/Services/AppInfo/X-TRACT.aspx) software was used for computed axial tomography (CT) reconstruction following a Feldkamp-Davis-Kress (FDK)-based algorithm [27]. The reconstructed volumes were smoothed over 2 voxels in the vertical direction to reduce the effect of a residual line-by-line misalignment due to hysteretic response of the scanning system to the rapid, bi-directional scanning pattern employed. The three-dimensional (3-D) elemental reconstructions preserved the quantitative nature of the measurement, with voxel values representing volumetric concentrations. These reconstructed volumes can be interrogated to determine volumetric elemental co-localization, scatter plots, and linear profile concentration gradients. 3-D rendering was performed using Avizo software (ver. 6.2, VSG).

## Supporting Information

Figure S1 Stereo view of elements in adult *C. elegans*. Shown are K (white), Ca (yellow), Mn (red), Fe (green) and Zn (blue).
(PDF)

Movie S1 Stereo view and movie of a tomographic reconstruction of the elemental content in *C. elegans*.
(MP4)
