# Supplementary figures and images for: Caenorhabditis elegans Maintains Highly Compartmentalized Cellular Distribution of Metals and Steep Concentration Gradients of Manganese
Source: PLoS One. 2012 Feb 29;7(2):e32685. doi: 10.1371/journal.pone.0032685 (PMC3290589; doi:10.1371/journal.pone.0032685)

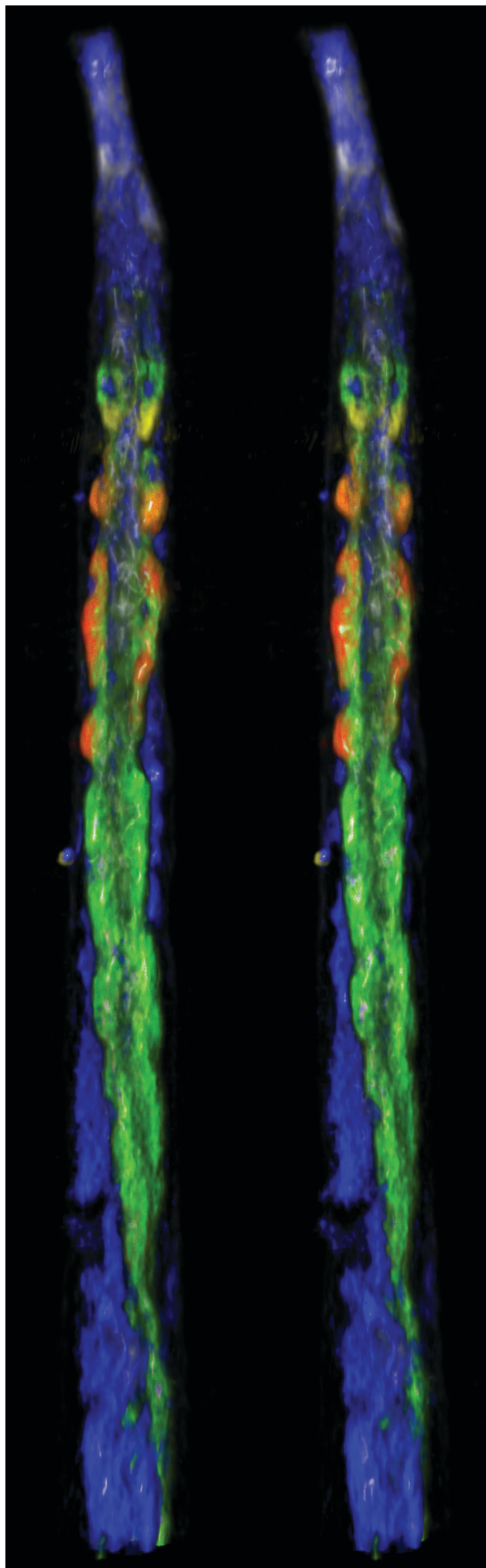

Fig S2.

Supplement: Figure S1 — Stereo view of elements in adult C. elegans. Shown are K (white), Ca (yellow), Mn (red), Fe (green) and Zn (blue). (PDF) [file pone.0032685.s001.pdf]
